# A Practical Application of Machine Learning for the Development of Metallole-Based Fluorescent Materials

**DOI:** 10.3390/molecules30081686

**Published:** 2025-04-10

**Authors:** Yusuke Kanematsu, Akiyoshi Ohta, Shunya Nagai, Yohei Adachi, Hiromasa Kaneko, Takayoshi Ishimoto, Takio Kurita, Joji Ohshita

**Affiliations:** 1 Smart Innovation Program, Graduate School of Advanced Science and Engineering, Hiroshima University, Higashi-Hiroshima 739-8527, Japanyadachi@hiroshima-u.ac.jp (Y.A.); hkaneko@meiji.ac.jp (H.K.); tishimo@hiroshima-u.ac.jp (T.I.); 2 Division of Materials Model-Based Research, Digital Monozukuri (Manufacturing) Education and Research Center, Hiroshima University, Higashi-Hiroshima 739-0046, Japan; 3 Department of Applied Chemistry, School of Science and Technology, Meiji University, Kawasaki 214-8571, Japan; 4 (Professor Emeritus) Informatics and Data Science Program, Graduate School of Advanced Science and Engineering, Hiroshima University, Higashi-Hiroshima 739-8527, Japan; tkurita@hiroshima-u.ac.jp

**Keywords:** fluorescent materials, machine learning, model-based research

## Abstract

We have built a prediction model of the fluorescence quantum yields of metalloles. Based on the suggestion by the prediction model, we synthesized 10 fluorescent molecules to confirm the prediction accuracy. By measuring the fluorescence quantum yields of the synthesized molecules, it was demonstrated that our prediction model reasonably classified the quantum yields with an accuracy of 0.7. In particular, the low quantum yields were perfectly predicted for the synthesized molecules, demonstrating the usefulness of our prediction model to screen out weakly fluorescent molecules from the candidates. On the other hand, the low precision of 0.5 was attributed to the bias in the training dataset containing many fluorine-containing molecules with high quantum yields. Our prediction model was then revised with the generator of candidate molecular structures for more efficient development of fluorescent materials with taking the applicability domain into account, and the improvement of the applicability was confirmed owing to the increment of the dataset.

## 1. Introduction

**Figure 1:**
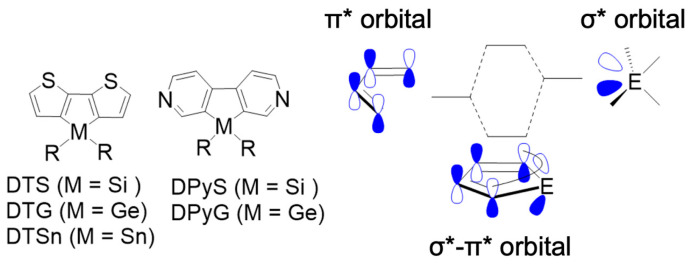
Metallole-based molecular skeletons with group 14 elements and the σ*-π* conjugation.

Organic luminescent compounds have been extensively studied for use as imaging, sensing, and electroluminescent materials. To enable practical applications, the control of quantum efficiencies of photoluminescence as well as absorption and luminescence wavelengths is of utmost importance. However, in contrast to the fact that absorption and luminescence wavelengths may be predicted by high-level computer simulations of molecular orbitals, the prediction of photoluminescence quantum yield remains difficult. This is because many factors complicatedly affect the quantum yield, including molecular rigidity, probability of inter-system crossing, and relaxation and electronic states of the photoexcited molecules. Metallole is a derivative of cyclopentadiene whose sp^3^ carbon atom is replaced by an inorganic heteroatom [1]. Silole and germole, two metalloles whose heteroatoms are Si and Ge, respectively, are known to possess low LUMO energy levels owing to the strong interaction between the π* orbital of cyclopentadiene and the σ* orbital of Si [2] and Ge [3] atoms (Figure 1). Aromatic ring-fused silole and germole, dithienosilole (DTS) and dithienogermole (DTG), respectively, are of particular interest because they usually show high photoluminescence quantum yields owing to the fact that they have sufficient rigidity to reduce the nonradiative deactivation of the photoexcited states [4]. Another important aspect of the DTS and DTG scaffolds is the extensibility by using such groups as aryl [5] and polysilsesquioxanes (PSQ) [6]; their optical properties including the quantum yields can be significantly modulated by substituents that can be readily introduced at the reactive α-positions of the thiophene rings. Meanwhile, with the aid of π-π stacking, DTG derivatives were reported to form a helical assembly that revealed switchable circularly polarized luminescence (CPL) [7]. Therefore, DTS and DTG are promising molecular units for the development of fluorescent materials such as fluorescent biomarkers [8], gas sensors [9], and functional dyes [10]. However, the effect of the substituent on the quantum yield is generally unpredictable because of the complexity of the deactivation mechanism that has many possible pathways, as mentioned above, and hence it has been indispensable to perform experiments by trial and error to achieve the desired fluorescence.

Machine learning (ML) techniques have been increasingly utilized in recent years with the aim of reducing the enormous number of trial-and-error attempts in materials development [11]. The most common use of ML is to search for candidate materials/molecules with given properties by predicting the properties of unknown samples using data obtained through trial and error in the past for training the prediction model. Although the usefulness of such ML predictions has been verified even for the photoluminescence quantum yield in a previous study [12], to our knowledge, there are few reports of practical use in the field of organic synthesis of fluorescent materials [13,14]. With the recent expansion of open-source libraries related to computational science, the bar has been dramatically lowered for the implementation of ML for a specific purpose, and thus even non-specialists in computational science can easily familiarize themselves with ML. Therefore, we aimed to test its practicality in the field of organic synthesis by running a cycle of prediction, synthesis, and model reconstruction.

The present study is devoted to the demonstration of an ML-aided synthesis of fluorescent materials. We have built a prediction model of the fluorescence quantum yield (hereinafter “quantum yield”). Based on the suggestion by the prediction model, we synthesized 10 fluorescent molecules to confirm the prediction accuracy. The model was then revised to improve the efficiency of the cycle for ML-aided synthesis. Although there is still much room for improvement, we conclude that the present prediction model is useful for the screening of candidates for novel fluorescent materials.

## 2. Results and Discussion

### 2.1. Evaluation of the Classification Model

We built RF-2D, RF-3D^+^, LGBM-2D, and LGBM-3D^+^ classification models by using 2D and 3D descriptors. Their accuracies for the classification of quantum yields evaluated by CV are shown in Table 1, which measure their prediction performance. The accuracies for the training dataset are also shown in Table S1, which indicate their fitting capacity. It should be noted that whereas the accuracies can be higher than 0.9 for the training dataset as shown in Table S1 because of overfitting, the accuracies of CV are less than 0.8. Small improvements were achieved by the feature selection, where the number of the descriptors was reduced to 14 or less. This suggests that the majority of the initial descriptors had little correlation with the quantum yields, which may contribute only to the overfitting of the training dataset. 

For practical purposes, it is more important to predict correctly the high quantum yields than the low values. Precision is a more suitable measure for this purpose, which is the ratio of correct predictions among the subsets with predicted quantum yields higher than 0.5. To easily achieve the higher precision value, we built the CPM by combining the four classification models (RF-2D, RF-3D, LGBM-2D, and LGBM-3D), which returns 1 when all of the four models return 1, otherwise, it returns 0. The cross-validated accuracies and the precisions of the CPM and RF-3D model are shown in Table 2. As was intended, the precision was increased from 0.78 to 0.85 by the combination of the four classifiers, whereas the accuracy was decreased from 0.82 to 0.78. Therefore, we expected that the CPM would enable an efficient search for candidate molecules with the desired quantum yields.

### 2.2. Spectroscopy of New Molecules

**Figure 2:**
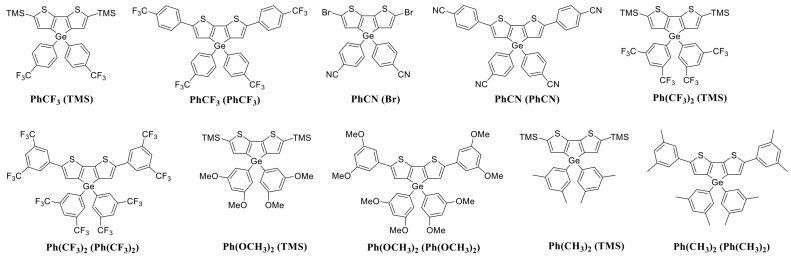
Newly synthesized DTG-based molecules.

Using the CPM discussed above, we made predictions and verified their accuracy by synthesizing 10 DTG-based molecules whose quantum yields have not been reported. The synthesized molecules are shown in Figure 2 and the predicted labels for them are listed in Table 3. 

**Figure 3:**
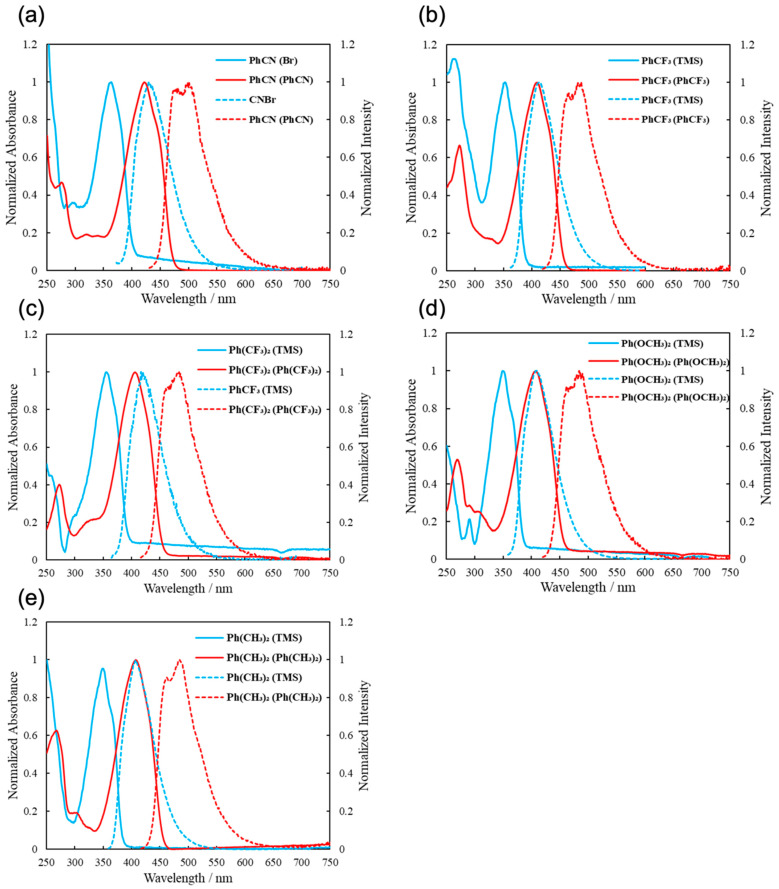
Absorption spectra (solid lines) and fluorescence spectra (dashed lines) of Ar^1^ (TMS), Ar^1^ (Br), and Ar^1^ (Ar^2^). Ar = (**a**) PhCN, (**b**) PhCF_3_, (**c**) Ph(CF_3_)_2_, (**d**) Ph (OCH_3_)_2_, and (**e**) Ph (CH_3_)_2_ in THF.

We then measured the absorption and fluorescence spectra of the synthesized molecules. In Figure 3, the solid and dashed lines represent the absorption and fluorescence spectra in THF, respectively. The absorption and emission wavelengths are summarized in Table 4. The corresponding quantum yields are discussed in the next section. By comparing Ar^1^(TMS), Ar^1^(Br), and Ar^1^(Ar^2^) molecules, the Ar^1^(Ar^2^) compounds were found to exhibit longer wavelength shifts of approximately 50 to 60 nm in the absorption spectra and 60 to 70 nm in the fluorescence spectra, respectively. This could be attributed to the extended π-conjugation resulting from the introduction of the π-conjugated substituents on thiophene. PhCN(PhCN) with cyano groups (C≡N) was found to show red shifts of approximately 10 nm in both absorption and fluorescence spectra, relative to the other Ar^1^(Ar^2^) compounds. This may be also attributed to the extended conjugation brought about by the cyano group. 

**Figure 4:**
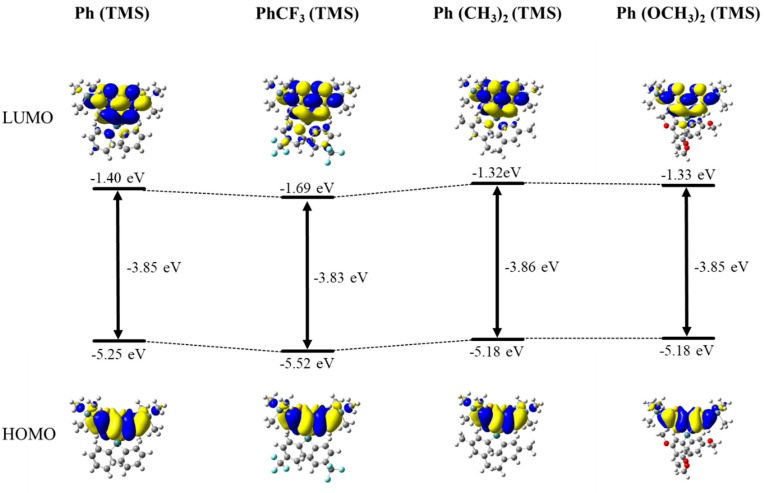
Frontier molecular orbitals and HOMO/LUMO energy levels of Ph(TMS), PhCF_3_(TMS), Ph(CH_3_)_2_ (TMS), and Ph(OCH_3_)_2_ (TMS) from DFT calculation (B3LYP/6-31G(d,p)).

Comparing the Ar^1^(TMS) molecules, those with the electron-withdrawing trifluoromethyl group (CF_3_) on the Ar group have absorption and fluorescence wavelengths that were red-shifted by a few nm relative to those with electron-donating substituents, methoxy group (OCH_3_) and methyl group (Me). We believe that this is because the effect of the introduction of CF_3_ on the LUMO energy level is larger than the effect of the introduction of OCH_3_ or CH_3_ on the HOMO energy level. As a result, molecules with CF_3_ are expected to have a smaller band gap and a longer wavelength shift than molecules with the other substituents (Figure 4). This effect is observed for Ar^1^(TMS) but not for Ar^1^(Ar^2^); this is probably because the introduction of an aryl group on the thiophene ring has a significant effect on the extension of the π-conjugation and the effect of the substituent on Ge is relatively small. 

### 2.3. Comparison of Observed and Predicted Quantum Yields

The observed and predicted quantum yields of the newly synthesized molecules are shown in Table 5. We can see that the prediction accuracy is 0.7; 7 of the 10 predictions are correct. Therefore, it can be said that the prediction model developed in this study was able to acquire high prediction accuracy by learning the correlation between the molecular structure and the quantum yield latent in the training dataset. For example, it is known empirically that the introduction of a silyl group into a π-conjugated system improves the quantum yield, and our prediction model correctly predicts high quantum yields of PhCF_3_(TMS) and Ph(CH_3_)_2_(TMS). Heavy atoms such as bromine promote spin-orbit interactions, and their introduction into a luminescent molecule increases the rate constant of the inter-system crossing and decreases the luminescence. Indeed, the quantum yield of PhCN(Br) is less than 2%, which was also correctly predicted. We can also see in Table 5 that the four predicted low QYs were all correct, demonstrating the usefulness of our prediction model to screen out weakly fluorescent molecules from the candidates in the process of searching for strongly fluorescent molecules. 

Whereas the accuracy was high, the precision that measures the correctness of the prediction of the high quantum yield failed to meet our expectations, with three out of six molecules. This result suggests that combining the four models to construct the CPM was not effective in increasing precision. The three molecules with the incorrect prediction were those in which the CF_3_ group was introduced. It has been reported that the introduction of fluorine into a molecule improves the quantum yield [15], and in the training dataset used in this study, approximately 60% of the molecules with fluorine introduced into the DTG and DTS skeletons had quantum yields higher than 60%. The CPM predicted that the quantum yield of Ph(CF_3_)_2_(Ph(CF_3_)_2_), in which fluorine was introduced into the low quantum yield molecule, Ph(CH_3_)_2_(Ph(CH_3_)_2_), would be higher, but in fact, the introduction of fluorine resulted in the decrement of the quantum yield. Given that all four of the fluorinated molecules were predicted to have high quantum yields, the prediction model seems to give monotonously high quantum yields for fluorinated DTG- and DTG-based molecules despite the fact that the introduction of fluorine can result in both the increment and decrement of quantum yields. It was found that the training dataset contained only two DTS derivatives with PhCF_3_ groups, both of which have quantum yields higher than 50%. Therefore, the overfitting problem was presumably due to a bias arising from the training dataset containing many fluorine-containing molecules with high quantum yields.

### 2.4. Revision of the Model with Extending Dataset

Whereas the availability of the ML-aided synthesis was practically confirmed with acceptable accuracy, there was concern regarding the monotonic overestimation of the quantum yield to reduce the prediction precision. It was also suggested that the combination of the classifiers may not contribute to the reduction in the number of false-positive predictions for fluorinated molecules. We therefore revised the prediction model by removing the redundancy in the building procedure to improve efficiency. The synthesized 10 molecules were added to the training dataset, and the prediction performance was evaluated by the test dataset that was split from the entire dataset, as shown in Table S1. The model revision reduced the time required to build a model by more than half. Table 6 shows the accuracy and precision of the revised models, Rev-RF and Rev-LGBM, and also those of the previous CPM for the training and test datasets. We can see that the performance of Rev-LGBM was comparable to that of the CPM, suggesting that the combination of the classifiers that increase the computational time could be dispensed with. We then focused on the DTS and DTG analogs in the test dataset. Table 7 shows the observed and predicted QY labels for 21 DTS and DTG analogs contained in the test dataset, including five fluorinated ones shown in gray. The precision for the subset was 1 for both the CPM and Rev-LGBM, whereas the total number of QY = 1 predictions was 2 and 4, respectively. Therefore, Rev-LGBM could be regarded as more effective for the screening of candidates than the CPM in this case. The predicted labels by the CPM for the five fluorinated molecules were monotonically 0, whereas the previous results for the CPM in Table 5 were monotonically 1. Given that such kind of monotonic prediction was not seen for Rev-LGBM, we concluded that Rev-LGBM would be the more reasonable choice of the prediction model for the screening of strongly fluorescent DTG- or DTS-based molecules. Although the present model is a simplistic binary classifier that does not allow quantitative prediction of quantum yield, the regression model using LightGBM will be available by increasing the training dataset and employing the descriptors obtained by density functional theory (DFT) such as relative energy levels of the frontier molecular orbitals, whose significance has been demonstrated for the photoinduced electron transfer [16].

## 3. Materials and Methods

### 3.1. Computational Details

We applied several open-source Python 3.11 packages in this study: Scikit-learn 1.6.1 [17] to build the ML models, RDKit 2019.9.1 [18] to obtain molecular descriptors as explanatory variables, LightGBM 4.6.0 (LGBM) [19] for the binary classification, and the Structure Generator based on R-Group (SGRG) [20] to generate the candidate molecules for the next synthesis. LightGBM is a lightweight and reliable model, whose usefulness has been confirmed in the previous applications for the development of organic fluorescent materials [14,21]. 

For ML in this study, we collected dataset of quantum yields of 369 fluorescent molecules registered in SciFinder as of March 2019. The distribution of the quantum yields in the dataset is shown in Figure S3, 161 (43.6%) of which are higher than 50%. The corresponding molecular structural information was provided as the Simplified Molecular Input Line Entry System (SMILES) representation [22]. The additional data of 19 molecules that have been synthesized in our laboratory were used to extend the dataset.

We utilized the 2D and 3D descriptors available in RDKit as the explanatory variables, whose details can be found on the RDKit website (https://www.rdkit.org/docs/GettingStartedInPython.html#list-of-available-descriptors, accessed on 5 February 2025). We also used the 2D descriptors included in the “descList” and “BCUT2D” objects of RDKit. For the calculation of 3D descriptors, 3D molecular structures were generated by using the “EmbedMolecule” function. We added frontier orbital energies and their gaps into the 3D descriptors calculated by the extended Hückel method implemented in the “rdEHTTools” library in RDKit. In total, 208 2D descriptors and 21 3D descriptors were available, although 3D descriptors were not available for some molecules with complicated structures because of the difficulty of 3D structure generation. 

#### 3.1.1. Building Initial Models

Several prediction models with Random Forest (RF) and LGBM classifiers were built, which classify whether a target molecule has a quantum yield that is higher than 50% or not, whose class label is defined as 1 (True) and 0 (False), respectively. Two-dimensional descriptors were available for all of the 363 entries in the present dataset, 344 of which were available for the Three-dimensional descriptor calculation. Therefore, RF and LGBM classifiers were trained for the 363 dataset with 2D descriptors and the 344 dataset with both 2D and 3D descriptors (hereinafter abbreviated as 3D^+^), which are named RF-2D, RF-3D^+^, LGBM-2D, and LGBM-3D^+^. It should be noted that the number of available datasets for 3D^+^ was decreased mainly because of failure to build a 3D structure from SMILES for some molecules. According to the cross validation (CV), we tuned the hyper parameter “n_estimators” 27 and “max_depth” 6 for RF, and “max_depth” 7 for LGBM to reduce the overfitting to the training dataset. However, the improvements in the score were marginal, and thus this tuning was omitted. In order to simply reduce the number of false-positive predictions, we built a simple combined prediction model (CPM) with these four classifiers. The CPM returns 1 only when all of RF-2D, RF-3D^+^, LGMB-2D, and LGBM-3D^+^ return 1; therefore, both the true- and false-positive prediction can be reduced. The prediction performance of the models was evaluated on the basis of the accuracy of the five-fold CV, which is the ratio of correct predictions. We performed the feature selection in two steps to simplify the model. First, we used the Recursive Feature Elimination (RFE) method implemented in Scikit-learn to reduce the number of descriptors to 18 for the LGBM model and to 17 for the RF model. We then exhaustively searched the combination of the descriptors that maximize the accuracy of CV.

#### 3.1.2. Model Revision

To enhance the efficiency of the cycle for ML-aided synthesis, we revised our classification models. We built the models named Rev-RF and Rev-LGBM according to the following procedure. We divided 363 data into a training dataset of 300 and a test dataset of 63 as shown in Table S2, the latter of which was used to evaluate the performance of the prediction. The percentages of samples with quantum yield higher than 50% are 44% and 40% for the training and test datasets, respectively. The hypermeters of the RF and LGBM classifiers were optimized by CV with the 300 training data, as shown in Table S3. We used the RFE method alone for the feature selection. Note that this RFE step may be dispensable since the improvement in the cross-validated accuracy of LGBM for the training dataset from 0.75 to 0.76 was very small. We checked whether the test dataset is within the applicability domain (AD) [23,24,25] of the model or not, to confirm the reliability of the prediction. The criterion for AD was calculated according to the following equation by the k-nearest neighbor (kNN) method [26] implemented in Scikit-learn,

(1) $$D_{crit}=\frac{1}{N}\sum_{i}^{N}{\bar{D}}_{5,i}+3\sigma$$

where ${\bar{D}}_{5,i}$ is the mean distance from the nearest 5 training data for data point *i*, the summation runs over all entries of the training dataset, and σ is the standard deviation of ${\bar{D}}_{5,i}$ within the training dataset. If ${\bar{D}}_{5,i}$. for some data is greater than $D_{crit}$, then the corresponding molecule would be regarded as an outlier from AD, and thus the prediction for the data as unreliable. In order to reduce the number of false positives of the predictions, Rev-EPM returns 1 only when both Rev-RF and Rev-LGBM return 1 and ${\bar{D}}_{5,i}$. is less than $D_{crit}$; otherwise, it returns 0.

### 3.2. Synthesis

**Scheme 1:**
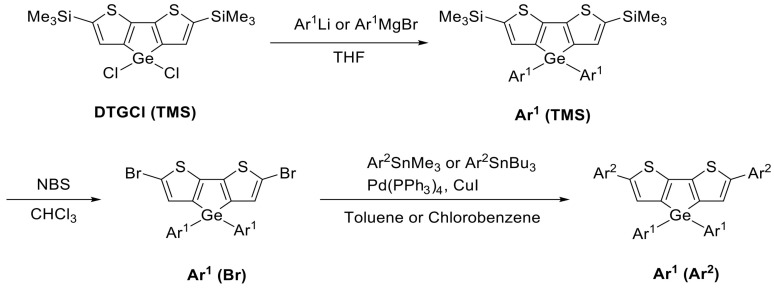
Synthesis of new fluorescent molecules.

New DTG derivatives were prepared as outlined in Scheme 1, and the details are available in supplementary materials. In the first step, DTGCl (TMS) was synthesized as reported in the literature [27] and reacted with Ar^1^Li or Ar^1^MgBr to obtain white solid Ar^1^(TMS). The Ar^1^(Br) derivative was then synthesized by the reaction with NBS. Finally, the Stille coupling reaction of Ar^1^(Br) with Ar^2^SnMe_3_ or Ar2SnBu_3_ gave the desired Ar^1^(Ar^2^) (Scheme 1). The yields of the synthetic intermediates and the target products are summarized in Table 8.

## 4. Conclusions

We applied machine learning techniques to explore novel metallole-based fluorescent materials with the desired levels of quantum yields. We built binary classification models that classify whether a given molecule has a quantum yield higher than 50% or not. Cross validation of the classification models suggested reasonable accuracy for the prediction of the quantum yield, and that the combination of classifiers would slightly improve the precision of the prediction. The performance of the combined prediction model was then evaluated through actual synthesis with screening for the candidates to confirm the reasonable prediction accuracy of 0.70. In particular, the low quantum yields were perfectly predicted for the synthesized molecules, demonstrating the usefulness of our prediction model to screen out weakly fluorescent molecules from the candidates. On the other hand, the precision of 0.5 suggested that the present approach of combining similar classifiers was not so effective to reduce the number of false-positive predictions as the cross validation suggested. We examined samples for which the prediction failed and found that the lower precision would be mainly attributed to the bias of the training dataset, according to the false-positive prediction for fluorinated DTG-based molecules that are only present in a small fraction of the training dataset. 

To enhance the efficiency of the cycle for ML-aided synthesis, we revised the classification models by adding data of the newly synthesized molecules into the dataset. The model building procedure was simplified, reducing the time required to build a model by more than half while maintaining the accuracy. Meanwhile, we found an improvement in the prediction precision for DTS and DTG analogs in the test dataset, which could be attributed to the additional data of the synthesized DTG-based molecules. Accordingly, the accuracy and the precision of the model will further improve by continuing the cycle of prediction and observation to expand available data.

## Figures and Tables

**Table 1:** Cross-validated accuracies of classification models before and after descriptor selection.

Classification Model	Accuracy (CV)		Number of Selected Descriptors
	Before Selection	After Selection	
RF-2D	0.75	0.81	14
RF-3D^+^	0.77	0.82	11
LGBM-2D	0.76	0.83	9
LGBM-3D^+^	0.76	0.83	10

**Table 2:** Cross-validated accuracies and precisions of RF-3D model and CPM.

Prediction Model	Accuracy (CV)	Precision (CV)
RF-3D^+^	0.82	0.78
CPM	0.78	0.85

**Table 3:** Predicted labels (QY) for 10 new fluorescent DTG molecules indicating whether they have quantum yields higher than 50% (1) or not (0).

Compound	Predicted QY	Compound	Predicted QY
PhCF_3_ (TMS)	1	Ph (CF_3_)_2_ (Ph (CF_3_)_2_)	1
PhCF_3_ (PhCF_3_)	1	Ph (OCH_3_)_2_ (TMS)	1
PhCN (Br)	0	Ph (OCH_3_)_2_ (Ph (OCH_3_)_2_)	0
PhCN (PhCN)	0	Ph (CH_3_)_2_ (TMS)	1
Ph(CF_3_)_2_ (TMS)	1	Ph (CH_3_)_2_ (Ph (CH_3_)_2_)	0

**Table 4:** Absorption (*λ*_abs_ in nm) and emission (*λ*_em_ in nm) wavelengths measured for Ar^1^ (TMS), Ar^1^ (Br), and Ar^1^ (Ar^2^) in THF.

Compound	*λ*_abs_	*λ*_em_
PhCF_3_ (TMS)	353	414
PhCF_3_ (PhCF_3_)	409	487
PhCN (Br)	363	430
PhCN (PhCN)	421	499
Ph(CF_3_)_2_ (TMS)	355	417
Ph(CF_3_)_2_ (Ph (CF_3_)_2_)	406	484
Ph(OCH_3_)_2_ (TMS)	349	409
Ph(OCH_3_)_2_ (Ph (OCH_3_)_2_)	407	484
Ph(CH_3_)_2_ (TMS)	349	407
Ph(CH_3_)_2_ (Ph (CH_3_)_2_)	407	485

**Table 5:** Fluorescence lifetimes (*τ* in ns), quantum yields (*Φ*_f_ in %) in THF solvent, and binary labels of quantum yield (QY) for newly synthesized molecules. The predicted QYs are the same as those in Table 3.

Compound	Observed			Predicted
	*τ*	*Φ*_f_	QY	QY
PhCF_3_ (TMS)	4.25	70	1	1
PhCF_3_ (PhCF_3_)	1.26	40	0	1
PhCN (Br)	1.13	<2	0	0
PhCN (PhCN)	0.81	18	0	0
Ph(CF_3_)_2_ (TMS)	4.62	46	0	1
Ph(CF_3_)_2_ (Ph (CF_3_)_2_)	1.35	28	0	1
Ph(OCH_3_)_2_ (TMS)	3.88	58	1	1
Ph(OCH_3_)_2_ (Ph (OCH_3_)_2_)	1.42	29	0	0
Ph(CH_3_)_2_ (TMS)	3.42	71	1	1
Ph(CH_3_)_2_ (Ph (CH_3_)_2_)	1.18	42	0	0

**Table 6:** Accuracies and precisions of the revised models compared with the previous CPM.

Classification Model		Rev-RF	Rev-LGBM	CPM
Accuracy	Train	0.97	0.98	0.93
	Test	0.86	0.84	0.84
Precision	Train	0.94	0.96	1.00
	Test	0.81	0.86	0.89

**Table 7:** Results for 21 DTS or DTG analogs in test dataset. Fluorinated molecules are shaded in gray.

Molecular Index	Observed		Predicted QY		
	*Φ*_f_	QY	CPM	Rev-RF	Rev-LGBM
1	70	1	0	1	1
2	84	1	1	1	1
3	0	0	0	0	0
4	2	0	0	0	0
5	7	0	0	0	0
6	20	0	0	0	0
7	13	0	0	0	0
8	62	1	0	1	1
9	71	1	1	1	1
10	9	0	0	0	0
11	5	0	0	0	0
12	17	0	0	0	0
13	25	0	0	0	0
14	54	1	0	1	0
15	35	0	0	0	0
16	25	0	0	1	0
17	67	1	0	0	0
18	8	0	0	0	0
19	22	0	0	0	0
20	10	0	0	0	0
21	18	0	0	0	0
Accuracy			0.81	0.90	0.90
Precision			1.00	0.83	1.00

**Table 8:** Yields of Ar^1^ (TMS), Ar^1^ (Br), and Ar^1^ (Ar^2^).

Ar (Ar^1^ = Ar^2^)	Ar^1^ (TMS)/%	Ar^1^ (Br)/%	Ar^1^ (Ar^2^)/%
PhCF_3_	26	91	28
PhCN	34	87	21
Ph(CF_3_)_2_	46	70	16
Ph(OCH_3_)_2_	33	73	17
Ph(CH_3_)_2_	74	75	19

## Data Availability

The data presented in this study are available upon request from the corresponding author.

## Supplementary Materials

The following supporting information can be downloaded at: https://www.mdpi.com/article/10.3390/molecules30081686/s1, Figure S1: Result of classification for fluorescent molecules. Details of Rev-RF, (a) train, (b) test and Rev-LGBM (c) train, (d) test.; Figure S2: ^1^H NMR spectra for S1.12 to S1.16 compounds; Figure S3: Distribution of quantum yield of 369 molecules in the dataset; Table S1: Accuracy of the classification models for the training dataset before and after the selection of the descriptors; Table S2: Contents of the reconstructed training and test dataset; Table S3: The optimized hyperparameters for Rev-RF and Rev-LGBM models. The feature selection was performed by using RFE; The dataset excel file is also available as a supplementary material [27].

## Abbreviations

The following abbreviations are used in this manuscript:

CV	Cross validation
DTG	Dithienogermole
DTS	Dithienosilole
LGBM	Light Gradient Boosting Machine
RF	Random Forest
