# Supplementary material for: A Practical Application of Machine Learning for the Development of Metallole-Based Fluorescent Materials
Source: Molecules. 2025 Apr 10;30(8):1686. doi: 10.3390/molecules30081686 (PMC12029446; doi:10.3390/molecules30081686)
Supplement: Supplementary file 1 [file molecules-30-01686-s001.zip › Supplementary Files/SI_PLQY.rev.5_molecules.docx]

**Supporting Information**

**A Practical Application of Machine Learning for the Development of Metallole-based Fluorescent Materials**

Yusuke Kanematsu,^1,2,^* Akiyoshi Ohta,^1^ Shunya Nagai,^1^ Yohei Adachi,^1^ Hiromasa Kaneko,^1,3^ Takayoshi Ishimoto,^1,2^ Takio Kurita,^4^ Joji Ohshita^1,2,^*

^1^ Smart Innovation Program, Graduate School of Advanced Science and Engineering, Hiroshima University, Higashi-Hiroshima 739-8527, Japan; ykanem@hiroshima-u.ac.jp

^2^ Division of Materials Model-Based Research, Digital Monozukuri (Manufacturing) Education and Research Center, Hiroshima University, 3-10-32 Kagamiyama, Higashi-Hiroshima, Hiroshima 739-0046, Japan; [jo@hiroshima-u.ac.jp](mailto:jo@hiroshima-u.ac.jp)

^3^ Department of Applied Chemistry, School of Science and Technology, Meiji University, Kawasaki, Kanagawa 214-8571, Japan; [hkaneko@meiji.ac.jp](mailto:hkaneko@meiji.ac.jp)

^4^ (Professor Emeritus) Informatics and Data Science Program, Graduate School of Advanced Science and Engineering, Hiroshima University, Higashi-Hiroshima 739-8527, Japan; tkurita@hiroshima-u.ac.jp

**S1. Details of synthesis.**

**S1.1 General**

All reactions were carried out in dry argon. THF, diethyl ether, and toluene, and chlorobenzene used as the reaction solvents were distilled from calcium hydride and phosphorus pentoxide, respectively. They were stored over activated molecular sieves (4A) in dark until use. **DTGCl(TMS)** was prepared as reported in the literature.^1^ ^1^H and ^13^C NMR spectra were measured on Varian 400MR and system500 spectrometers. ESI and APCU mass spectra were measured on a Thermo Fisher Scientific LTQ Orbitrap XL spectrometer at N-BARD, Hiroshima University. UV-vis absorption and photoluminescence (PL) spectra were measured on Hitachi U-2910 and HORIBA FluoroMax-4 spectrophotometers, respectively. PL quantum yields were determined on a HORIBA FluoroMax-4 spectrofluorometer using a calibrated integrating sphere system.

**S1.2 Preparation of PhCF_3_(TMS)**

To a mixture of 0.120 g (4.94 mmol) of magnesium and 2 mL of THF was added dropwise a solution of 0.65 mL (4.65 mmol) of *p*-bromo(trifluoromethyl)benzene in 8 mL of THF and the mixture was stirred at room temperature for 2 h. The resultant mixture containing *p*-(trifluoromethyl)phenylmagnesium bromide was added slowly to a solution of 1.00 g (2.21 mmol) of **DTGCl(TMS)** in 10 mL of THF and the mixture was heated to reflux overnight. The mixture was cooled to room temperature and hydrolyzed with aqueous ammonium chloride. The organic layer was separated and the aqueous layer was extracted with hexane. The organic layers were combined and washed with water then with brine. After drying over anhydrous magnesium sulfate, the solvents were evaporated and the residue was subjected to silica gel column chromatography to give 380 mg (26 %) of **PhCF_3_(TMS)** as white solids: ^1^H NMR (400 MHz, CDCl_3_) δ: 7.69 (2H, d, *J* = 7.8 Hz, Ph), 7.63 (2H, d, *J* = 7.8 Hz, Ph), 7.25 (2H, s, Th), 0.34 (18H, s, SiMe_3_). ^13^C NMR (101 MHz, CDCl_3_) δ: 153.5, 143.1, 140.4, 138.8, 135.8, 135.0, 132.1 (q, *J* = 32 Hz), 125.2 (q, *J* = 3.7 Hz), 124.0 (q, *J* = 272 Hz), 0.1. HRMS (APCI) Calcd for C_28_H_28_F_6_GeS_2_Si_2_ [M^+^]: 672.02814, Found: 672.02936. mp: 171-173 ºC.

**S1.3 Preparation of PhCN (TMS)**

To a solution of 0.850 g (4.64 mmol) of *p*-benzonitrile in 40 mL of THF was added dropwise 3.00 ml (4.64 mmol) of a 1.55 M hexane solution of *n*-butyllithium at -80 ºC and the mixture was stirred for 30 min at this temperature. To this was added 1.02 g (2.26 mmol) of **DTGCl(TMS)** and the mixture was allowed to warm to room temperature. After hydrolysis with aqueous ammonium chloride, the organic layer was separated and the aqueous layer was extracted with chloroform. The organic layers were combined and washed with water then with brine. After drying over anhydrous magnesium sulfate, the solvents were evaporated and the residue was subjected to silica gel column chromatography to give 447 mg (47 %) of **PhCN(TMS)** as white solids: ^1^H NMR (400 MHz, CDCl_3_) δ: 7.66 (8H, s, Ph), 7.23 (2H, s, Th), 0.34 (18H, s, SiMe_3_). ^13^C NMR (101 MHz, CDCl_3_) δ: 153.6, 143.5, 140.3, 139.4, 135.5 (m), 135.1, 131.8, 118.4, 113.8, 0.0. HRMS (APCI) Calcd for C_28_H_28_N_2_GeS_2_Si_2_ [M^+^]: 586.04387, Found: 586.04517. mp: 189-192 ºC.

**S1.4 Preparation of Ph(CF_3_)_2_(TMS)**の合成

To a mixture of 0. 131 g (5.39 mmol) of magnesium and 2 mL of THF was added dropwise a solution of 1.44 g (4.65 mmol) of 1-bromo-3,5-bis(trifluoromethyl)benzene in 8 mL of THF and the mixture was stirred at room temperature for 30 min. The resultant mixture containing 3,5-bis(trifluoromethyl)phenylmagnesium bromide was added slowly to a solution of 1.02 g (2.26 mmol) of **DTGCl(TMS)** in 4 mL of THF and the mixture was heated to reflux overnight. The mixture was cooled to room temperature and hydrolyzed with aqueous ammonium chloride. The organic layer was separated and the aqueous layer was extracted with ethyl acetate. The organic layers were combined and washed with water then with brine. After drying over anhydrous magnesium sulfate, the solvents were evaporated and the residue was subjected to silica gel column chromatography to give 820 mg (46 %) of **Ph(CF_3_)_2_(TMS)** as white solids: ^1^H NMR (400 MHz, CDCl_3_) δ: 7.94 (6H, br s, Ph), 7.27 (2H, s, Th), 0.36 (18H, s, SiMe_3_). ^13^C NMR (101 MHz, CDCl_3_) δ: 154.0, 144.3, 138.2, 136.7, 135.3, 134.4 (m), 131.9 (q, *J* = 33.4 Hz), 124.3 (m), 123.2 (q, *J* = 273 Hz), 0.0. HRMS (APCI) Calcd for C_30_H_26_F_12_GeS_2_Si_2_ [M^+^]: 808.00291, Found: 808.00421. mp: 151-153 ºC.

**S1.5 Preparation of Ph(OCH_3_)_2_(TMS)**

To a solution of 1.06 g (4.87 mmol) of 1-bromo-3,5-dimethoxybenzene in 10 mL of THF was added dropwise 3.10 ml (4.93 mmol) of a 1.59 M hexane solution of *n*-butyllithium at -80 ºC and the mixture was stirred for 30 min at this temperature. To this was added 1.01 g (2.23 mmol) of **DTGCl(TMS)** and the mixture was allowed to warm to room temperature. After hydrolysis with aqueous ammonium chloride, the organic layer was separated and the aqueous layer was extracted with chloroform. The organic layers were combined and washed with water then with brine. After drying over anhydrous magnesium sulfate, the solvents were evaporated and the residue was subjected to silica gel column chromatography to give 474 mg (33 %) of **Ph(OCH_3_)_2_(TMS)** as white solids:

^1^H NMR (400 MHz, CDCl_3_) δ: 7.26 (2H, s, Th), 6.72 (4H, d, *J* = 2.3 Hz, Ph), 6.48 (2H, t, *J* = 2.3 Hz), 3.75 (12H, s, OCH_3_), 0.33 (18H, s, SiMe_3_). ^13^C NMR (101 MHz, CDCl_3_) δ: 160.7, 153.1, 142.1, 141.9, 136.3, 136.2, 112.4, 101.4, 55.3, 0.1. HRMS (APCI) Calcd for C_30_H_38_O_4_GeS_2_Si_2_ [M^+^]: 656.09563, Found: 656.09692. mp: 153-154 ºC.

**S1.6 Preparation of Ph(CH_3_)_2_(TMS)**

To a solution of 1.66 g (8.97 mmol) of 1-bromo-3,5-dimethylbenzene in 20 mL of THF was added dropwise 5.80 ml (9.22 mmol) of a 1.59 M hexane solution of *n*-butyllithium at -80 ºC and the mixture was stirred for 90 min at this temperature. To this was added 1.90 g (4.20 mmol) of **DTGCl(TMS)** and the mixture was allowed to warm to room temperature. After hydrolysis with aqueous ammonium chloride, the organic layer was separated and the aqueous layer was extracted with ethyl acetate. The organic layers were combined and washed with water then with brine. After drying over anhydrous magnesium sulfate, the solvents were evaporated and the residue was subjected to silica gel column chromatography to give 1.83 g (74 %) of **Ph(CH_3_)_2_(TMS)** as white solids: ^1^H NMR (500 MHz, CDCl_3_) δ: 7.26 (2H, s, Th), 7.17 (4H, br s, Ph), 7.02 (2H, br s, 2H), 2.29 (12H, br s, Ph-CH_3_), 0.33 (18H, s, SiMe_3_). ^13^C NMR (101 MHz, CDCl_3_) δ: 152.9, 142.8, 141.6, 137.8, 136.5, 134.3, 132.2, 131.4, 21.3, 0.1. HRMS (APCI) Calcd for C_30_H_38_GeS_2_Si_2_ [M^+^]: 592.11598, Found: 592.11578. mp: 146-147 ºC.

**S1.7 Preparation of PhCF_3_(Br)**

To a solution of 306 mg (0.460 mmol) of **PhCF_3_(TMS)** in 20 mL of chloroform was added174 mg (0.980 mmol) of NBS 174 mg (0.980 mmol) in several potions at 0 ºC. After stirring 10 min at this temperature, the mixture was allowed to warm to room temperature and further stirred for 90 min. An aqueous solution of sodium thiosulfate was added to the mixture and the organic layer was separated then the aqueous layer was extracted with chloroform. The organic layers were combined and washed with water then with brine. After drying over anhydrous magnesium sulfate, the solvents were evaporated to give 29.0 mg (91 %) of **PhCF_3_(Br)** as light yellow solids: ^1^H NMR (400 MHz, CDCl_3_) δ: 7.65 (8H, s, Ph), 7.14 (2H, s, Th). HRMS (APCI) Calcd for C_22_H_10_Br_2_F_6_GeS_2_ [M^+^]: 683.77012, Found: 683.77026. mp: 190-192 ºC.

**S1.8 Preparation of PhCN(Br)**

To a solution of 342 mg (0.584 mmol) of **PhCN(TMS)** in 13 mL of chloroform was added 223 mg (1.24 mmol) of NBS in several potions at 0 ºC. After stirring 10 min at this temperature, the mixture was allowed to warm to room temperature and further stirred for 5 h. An aqueous solution of sodium thiosulfate was added to the mixture and the organic layer was separated then the aqueous layer was extracted with chloroform. The organic layers were combined and washed with water then with brine. After drying over anhydrous magnesium sulfate, the solvents were evaporated to give 229 mg (87 %) of **PhCN(Br)** as light yellow solids: ^1^H NMR (400 MHz, CDCl_3_) δ: 7.68 (4H, d, *J* = 8.3 Hz, Ph), 7.61 (4H, d, *J* = 8.3 Hz, Ph), 7.13 (2H, s, Th). ^13^C NMR (101 MHz, CDCl_3_) δ: 148.2, 138.4, 136.7, 135.0, 132.1, 131.5 (m), 118.1, 114.4, 113.3. HRMS (APCI) Calcd for C_22_H_10_N_2_Br_2_GeS_2_ [M^+^]: 597.78584, Found: 597.78625. mp: 206-210 ºC.

**S1.9 Preparation of Ph(CF_3_)_2_(Br)**

To a solution of 690 mg (0.850 mmol) of **Ph(CF_3_)_2_(TMS)** in 20 mL of chloroform was added 348 mg (1.96 mmol) of NBS in several potions at 0 ºC. After stirring 5 min at this temperature, the mixture was allowed to warm to room temperature and further stirred for 3 h. An aqueous solution of sodium thiosulfate was added to the mixture and the organic layer was separated then the aqueous layer was extracted with chloroform. The organic layers were combined and washed with water then with brine. After drying over anhydrous magnesium sulfate, the solvents were evaporated. The residue was subjected to silica gel column chromatography to give 484 mg (70 %) of **Ph(CF_3_)_2_(Br)** as light yellow solids: ^1^H NMR (500 MHz, CDCl_3_) δ: 8.00 (2H, br s, Ph), 7.89 (4H, br s, Ph), 7.16 (2H, s, Th). ^13^C NMR (101 MHz, CDCl_3_) δ: 148.8, 135.4, 135.0, 134.1, 132.4 (q, *J* = 33.4 Hz), 131.3, 124.8 (m), 123.0 (q, *J* = 273 Hz), 114.1. HRMS (APCI) Calcd for C_24_H_8_Br_2_F_12_GeS_2_ [M^+^]: 819.74489, Found: 819.74652. mp: 204-206 ºC.

**S1.10 Preparation of Ph(OCH_3_)_2_(Br)**

To a solution of 340 mg (0.520 mmol) of **Ph(OCH_3_)_2_(TMS)** in 15 mL of chloroform was added 234 mg (1.31 mmol) of NBS in several potions at 0 ºC. After stirring 10 min at this temperature, the mixture was allowed to warm to room temperature and further stirred overnight. An aqueous solution of sodium thiosulfate was added to the mixture and the organic layer was separated then the aqueous layer was extracted with chloroform. The organic layers were combined and washed with water then with brine. After drying over anhydrous magnesium sulfate, the solvents were evaporated. The residue was subjected to silica gel column chromatography to give 253 mg (73 %) of **Ph(OCH_3_)_2_(Br)** as dark brown solids: ^1^H NMR (400 MHz, CDCl_3_) δ: 7.12 (2H, s, Th), 6.64 (4H, d, *J* = 2.3 Hz, Ph), 6.50 (2H, t, *J* = 2.3 Hz, Ph), 3.76 (12H, s, OCH_3_). HRMS (APCI) Calcd for C_24_H_20_O_4_Br_2_GeS_2_ [M^+^]: 667.83761, Found: 667.83832.

**S1.11 Preparation of Ph(CH_3_)_2_(Br)**

To a solution of 1.64 g (2.77 mmol) of **Ph(CH_3_)_2_(TMS)** in 30 mL of chloroform was added 1.38 mg (7.75 mmol) of NBS in several potions at 0 ºC. After stirring 10 min at this temperature, the mixture was allowed to warm to room temperature and further stirred for 1 h. An aqueous solution of sodium thiosulfate was added to the mixture and the organic layer was separated then the aqueous layer was extracted with chloroform. The organic layers were combined and washed with water then with brine. After drying over anhydrous magnesium sulfate, the solvents were evaporated and the residue was subjected to silica gel column chromatography to give 1.26 mg (75 %) of **Ph(CH_3_)_2_(Br)** as light yellow solids: ^1^H NMR (400 MHz, CDCl_3_) δ: 7.13 (2H, s, Th), 7.10 (4H, br s, Ph), 7.05 (2H, br s, Ph), 2.29 (12H, d, *J* = 0.6 Hz, Ph-CH_3_). ^13^C NMR (101 MHz, CDCl_3_) δ: 147.4, 140.1, 138.1, 132.6, 132.1, 131.94, 131.86, 112.0, 21.3. HRMS (APCI) Calcd for C_24_H_20_BrGeS_2_ [M^+^]: 603.85795, Found: 603.85754. mp.: 219-221 ºC.

**S1.12 Preparation of PhCF_3_(PhCF_3_)**

A mixture of 100 mg (0.146 mmol) of **PhCF_3_(Br)**, 115 mg (0.372 mmol) of *p*-(trifluoromethyl)(trimethylstannyl)benzene, 18.0 mg (15.6 μmol) of Pd(PPh_3_)_4_, 3.70 mg (19.4 μmol) of CuI, and 7 mL of toluene was heated to reflux overnight. The mixture was allowed to cool to room temperature and hydrolyzed with water. The organic layer was separated then the aqueous layer was extracted with chloroform. The organic layers were combined and washed with water then with brine. After drying over anhydrous magnesium sulfate, the solvents were evaporated and the residue was purified by silica gel column chromatography then by preparative GPC to give 33.0 mg (28%) of **PhCF_3_(PhCF_3_)** as yellow solids: ^1^H NMR (400 MHz, CDCl_3_) δ: 7.76–7.62 (16H, m, Ph), 7.51 (2H, s, Th). ^13^C NMR (101 MHz, CDCl_3_) δ: 148.3, 145.2, 140.4, 137.6, 137.4, 134.9, 126.6, 126.1 (m), 125.71, 125.5 (m). （低S/N比のためCF_3_及びC-CF_3_の4本が帰属できない。）　HRMS (APCI) Calcd for C_36_H_18_F_12_GeS_2_ [M^+^]: 815.98646, Found: 815.98743. mp: 262-263 ºC.

**S1.13 Preparation of PhCN(PhCN)**

A mixture of 149 mg (0.249 mmol) of **PhCN(Br)**, 189 mg (0.711 mmol) of *p*- (trimethylstannyl)benzonitrile, 15.3 mg (13.2 μmol) of Pd(PPh_3_)_4_, 9.80 mg (51.5 μmol) of CuI, and 8 mL of toluene was heated to reflux overnight. The mixture was allowed to cool to room temperature and hydrolyzed with water. The organic layer was separated then the aqueous layer was extracted with chloroform. The organic layers were combined and washed with water then with brine. After drying over anhydrous magnesium sulfate, the solvents were evaporated and the residue was subjected to silica gel column chromatography to give 45.0 mg (21%) of **PhCN(PhCN)** as orange solids:

^1^H NMR (400 MHz, CDCl_3_) δ: 7.70 (8H, s, Ph), 7.69 (8H, d, *J* = 2.4 Hz, Ph), 7.52 (2H, s, Th). HRMS (APCI) Calcd for C_36_H_18_N_4_GeS_2_ [M^+^]: 644.01792, Found: 644.01843. mp: > 300 ºC.

**S1.14 Preparation of Ph(CF_3_)_2_(Ph (CF_3_)_2_)**

A mixture of 201 mg (0.245 mmol) of **Ph(CF_3_)_2_(Br)**, 329 mg (0.654 mmol) of 3,5- bis(trifluoromethyl)(trimethylstannyl)benzene, 43.4 mg (37.6 μmol) of Pd(PPh_3_)_4_, 10.4 mg (54.6 μmol) of CuI, and 5 mL of chlorobenzene was heated to reflux overnight. The mixture was allowed to cool to room temperature and hydrolyzed with water. The organic layer was separated then the aqueous layer was extracted with chloroform. The organic layers were combined and washed with water then with brine. After drying over anhydrous magnesium sulfate, the solvents were evaporated and the residue was purified by silica gel column chromatography then by preparative GPC to give 42.0 mg (16%) of **Ph(CF_3_)_2_(Ph(CF_3_)_2_)** as yellow solids: ^1^H NMR (500 MHz, THF-*d*_8_) δ: 8.24 (s, 6H, Ph), 8.23 (s, 4H, Ph), 8.05 (s, 2H, Th), 7.98 (s, 2H, Ph).　13C NMRは純度が低すぎて帰属不可能HRMS (APCI) Calcd for C_40_H_14_F_24_GeS_2_ [M^+^]: 1087.93600, Found: 1087.93872. mp: 291-292 ºC.

**S1.15 Preparation of Ph(OCH_3_)_2_(Ph (OCH_3_)_2_)**

A mixture of 154 mg (0.230 mmol) of **Ph(OCH_3_)_2_(Br)**, 176 mg (0.654 mmol) of 3,5- dimethoxy(trimethylstannyl)benzene, 15.3 mg (13.2 μmol) of Pd(PPh_3_)_4_, 2.30 mg (12.1 μmol) of CuI, and 7 mL of chlorobenzene was heated to reflux overnight. The mixture was allowed to cool to room temperature and hydrolyzed with water. The organic layer was separated then the aqueous layer was extracted with chloroform. The organic layers were combined and washed with water then with brine. After drying over anhydrous magnesium sulfate, the solvents were evaporated and the residue was purified by silica gel column chromatography then by preparative GPC to give 30.0 mg (17%) of **Ph(OCH_3_)_2_(Ph(OCH_3_)_2_)** as yellow solids: ^1^H NMR (400 MHz, CDCl_3_) δ: 7.40 (2H, s, Th), 6.76 (4H, d, *J* = 2.2 Hz, Ph), 6.75 (4H, d, *J* = 2.3 Hz, Ph), 6.50 (2H, t, *J* = 2.3 Hz, Ph), 6.40 (2H, t, *J* = 2.2 Hz, Ph), 3.84 (12H, s, OCH_3_), 3.76 (12H, s, OCH_3_). ^13^C NMR (101 MHz, CDCl_3_) δ: 161.1, 160.9, 147.1, 145.9, 141.0, 136.2, 135.6, 125.9, 112.3, 104.0, 101.7, 99.8, 55.5, 55.4. HRMS (APCI) Calcd for C_40_H_38_O_8_GeS_2_ [M^+^]: 784.12144, Found: 784.12152. mp: 196-198 ºC.

**S1.16 Preparation of Ph(CH_3_)_2_(Ph(CH_3_)_2_)**

A mixture of 201 mg (0.245 mmol) of **Ph(CH_3_)_2_(Br)**, 329 mg (0.654 mmol) of 3,5- dimethyl(tributylstannyl)benzene, 43.4 mg (37.6 μmol) of Pd(PPh_3_)_4_, 10.4 mg (54.6 μmol) of CuI, and 7 mL of chlorobenzene was heated to reflux overnight. The mixture was allowed to cool to room temperature and hydrolyzed with water. The organic layer was separated then the aqueous layer was extracted with chloroform. The organic layers were combined and washed with water then with brine. After drying over anhydrous magnesium sulfate, the solvents were evaporated and the residue was purified by silica gel column chromatography then by preparative GPC to give 42.0 mg (16%) of **Ph(CH_3_)_2_(Ph(CH_3_)_2_)** as yellow solids: ^1^H NMR (400 MHz, CDCl_3_) δ: 7.41 (2H, s, Th), 7.25 (4H, br s, Ph), 7.21 (4H, br s, Ph), 7.04 (2H, br s, Ph), 6.91 (2H, br s, Ph), 2.35–2.33 (12H, m, Ph-CH_3_), 2.29 (12H, d, *J* = 0.5 Hz, Ph-CH_3_). ^13^C NMR (101 MHz, CDCl_3_) δ: 146.7, 145.8, 141.7, 138.4, 138.0, 134.5, 133.8, 132.2, 131.6, 129.0, 125.6, 123.5, 21.33, 21.31. HRMS (APCI) Calcd for C_40_H_38_GeS_2_ [M^+^]: 656.16212, Found: 656.16199. mp: 271-274 ºC.

**Table S1**. Accuracy of the classification models for the training dataset before and after the selection of the descriptors.

| Classification Model | Accuracy (Train) | | # of selected descriptors |
| --- | --- | --- | --- |
|  | Before selection | After selection |  |
| RF-2D | 0.94 | 0.94 | 14 |
| RF-3D | 0.94 | 0.91 | 11 |
| LGBM-2D | 1.00 | 0.99 | 9 |
| LGBM-3D | 1.00 | 0.98 | 10 |

**Table S2**. Contents of the reconstructed training and test dataset.

| Training dataset (300) | | Test dataset (63) | |
| --- | --- | --- | --- |
| Condition | # of data | Condition | # of data |
| *Φ*_f_ ≤ 50 | 168 | *Φ*_f_ ≤ 50 | 38 |
| *Φ*_f_ > 50 | 132 | *Φ*_f_ > 50 | 25 |

**Table S3**. The optimized hyperparameters for Rev-RF and Rev-LGBM models. The feature selection was done by using RFE.

| Hyper parameters | Rev-RF | Rev-LGBM |
| --- | --- | --- |
| n_estimators | 69 | 45 |
| max_depth | 6 | 5 |
| # of selected descriptors | 30 | 37 |


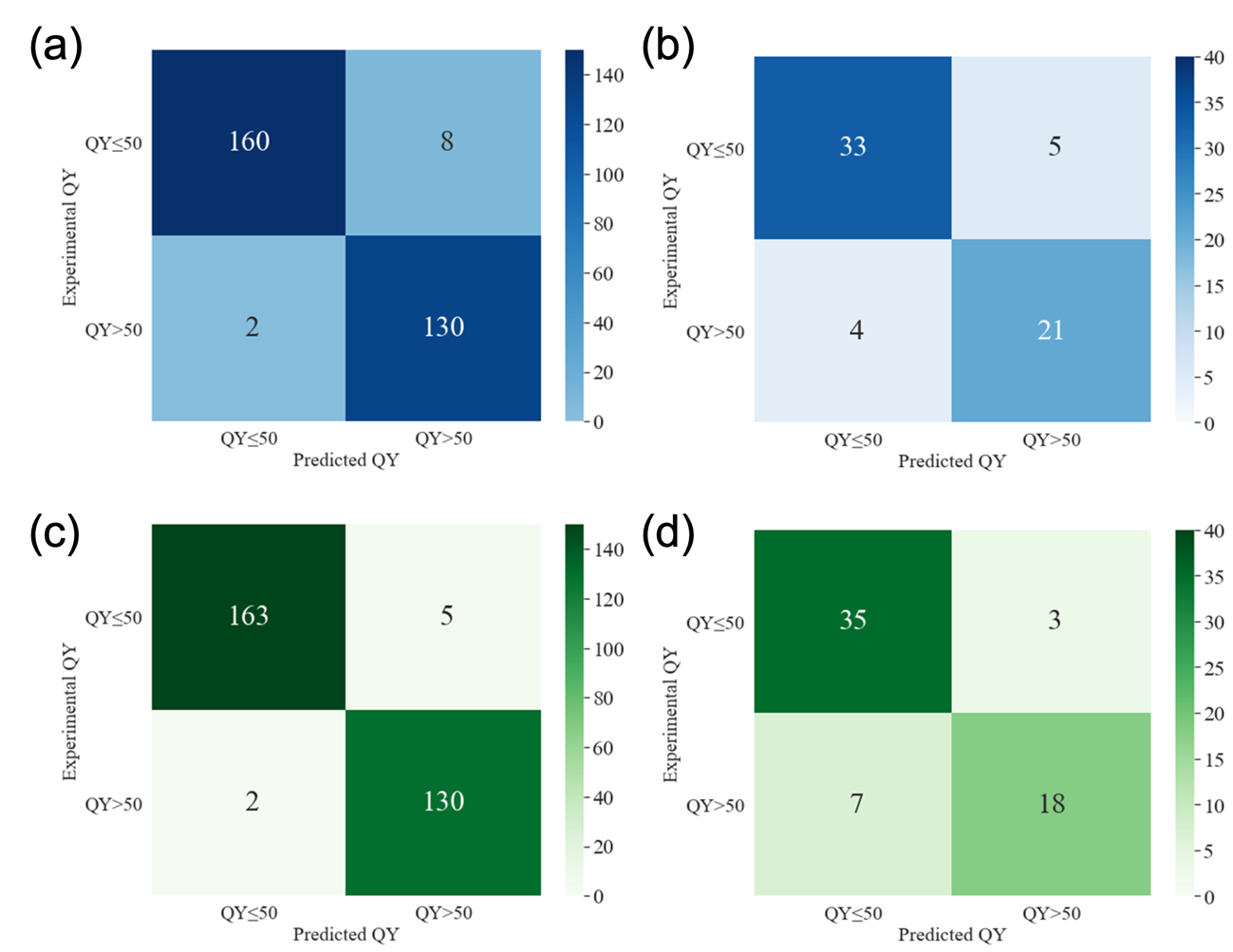


Figure S1. Result of classification for fluorescent molecules. Details of Rev-RF (a) Train, (b) Test, and Rev-LGBM (c) Train, (d) Test.


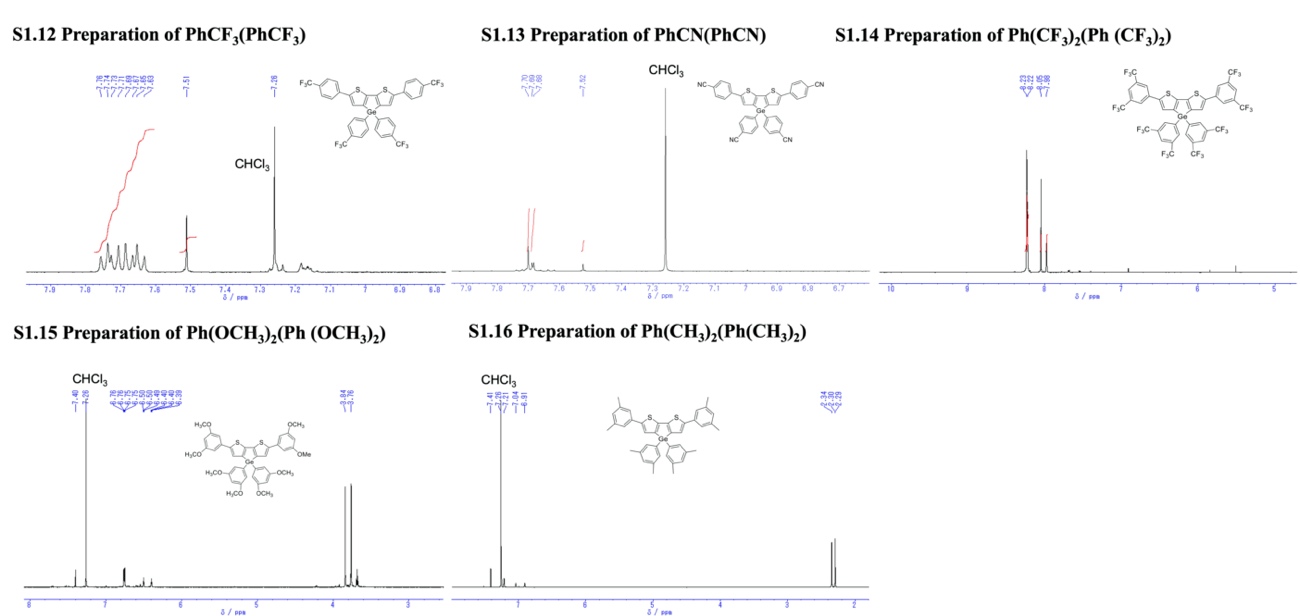


**Figure S2.** 1H NMR spectra for S1.12 to S1.16 compounds.


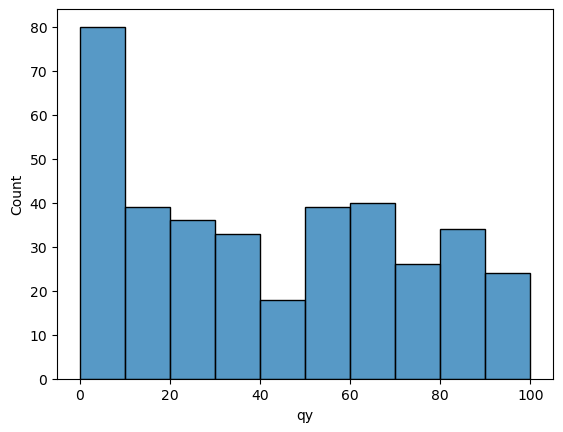


**Figure S3.** Distribution of the quantum yields of 369 molecules in the dataset.

**Reference**

1. Ohshita, J.; Nakamura, M.; Ooyama, Y. Preparation and Reactions of Dichlorodithienogermoles. *Organometallics* **2015**, *34*, 5609-5614.
